# Supplementary material for: Infer global, predict local: Quantity-relevance trade-off in protein fitness predictions from sequence data
Source: PLoS Comput Biol. 2023 Oct 26;19(10):e1011521. doi: 10.1371/journal.pcbi.1011521 (PMC10645369; doi:10.1371/journal.pcbi.1011521)
Supplement: S1 Text — Contains Appendices A and B. (PDF) [file pcbi.1011521.s001.pdf]

Supplementary Information

Infer global, predict local: quantity-relevance trade-off in protein fitness predictions from sequence data

Lorenzo Posani[1,2], Francesca Rizzato[1], Rémi Monasson[1], Simona Cocco[1]

1 Laboratory of Physics of the Ecole Normale Supérieure, CNRS UMR8023 & PSL Research, Sorbonne Université, 24 rue Lhomond, 75005 Paris, France.
2 Current address: Center for Theoretical Neuroscience, Columbia University, New York, NY 10027.
lorenzo.posani@gmail.com, simona.cocco@phys.ens.fr

Appendix A: approximate expression of the variance

Following [1, 2] we write an approximate expression for the variance σ_{ia}^2 based on the so-called 2-site approximation for the biases and couplings appearing in the K-links Potts model:

$$h_i(a) = \log p_i(a) \quad , \qquad J_{ij}(a,b) = \log\left(\frac{p_{ij}(a,b)}{p_i(a)\,p_j(b)}\right) \; . \tag{1}$$

These parameter values are exact for sparse interaction graphs with a tree-like structure.

We first detail the calculation in the $K = 0$ case (no coupling). Inserting Eq. (1) into Eq. 2 of the main text, we obtain

$$\widehat{\mathcal{E}}_{ia} = \log\left(\frac{p_i(a)}{p_i(wt_i)}\right) \; . \tag{2}$$

For the sake of simplicity, as site are independent, we can focus on one of them, and drop the i index. Each frequency p is stochastic as it varies with the sequence data, *i.e.* the sub-MSA. When the number of data, B, is large, the distribution of the frequencies is approximately Gaussian with

$$p(a) = p^*(a) + \frac{\epsilon(a)}{\sqrt{B}} \; , \tag{3}$$

where p^* are the ground-truth frequencies and the errors have first and second moments given by

$$[\epsilon(a)] = 0 \; , \quad [\epsilon(a)\,\epsilon(b)] = p^*(a)\,\delta_{a,b} - p^*(a)p^*(b) \; . \tag{4}$$

We may now expand $\widehat{\mathcal{E}}_{ia}$ to order $1/B$ according to (3), and compute its first and second moments, with the results:

$$\left[\widehat{\mathcal{E}}_{ia}\right] = \log\left(\frac{p^*(a)}{p^*(wt)}\right) - \frac{1}{2B}\left(\frac{[\epsilon(a)^2]}{p^*(a)^2} - \frac{[\epsilon(wt)^2]}{p^*(wt)^2}\right) \; , \tag{5}$$

$$\begin{aligned}\left[\widehat{\mathcal{E}}_{ia}^2\right] &= \log^2\left(\frac{p^*(a)}{p^*(wt)}\right) + \frac{1}{B}\left(\frac{[\epsilon(a)^2]}{p^*(a)^2} + \frac{[\epsilon(wt)^2]}{p^*(wt)^2} - 2\frac{[\epsilon(a)\epsilon(wt)]}{p^*(a)p^*(wt)}\right) \\ &- \frac{1}{B}\log\left(\frac{p^*(a)}{p^*(wt)}\right) \times \left(\frac{[\epsilon(a)^2]}{p^*(a)^2} - \frac{[\epsilon(wt)^2]}{p^*(wt)^2}\right)\end{aligned} \tag{6}$$

Substracting the two identities above and using the expression of the moments in Eq. (4) we obtain the following expression for the variance of $\widehat{\mathcal{E}}_{ia}$,

$$\sigma_{ia}^2 = \frac{1}{B}\left\{\frac{1}{p_i(a)} + \frac{1}{p_i(wt_i)}\right\} , \tag{7}$$

We now turn to the case of non-zero couplings ($K \neq 0$). The estimator of mutation cost now reads, according to Eqs. (1) and Eq. 2 of the main text,

$$\begin{aligned}\widehat{\mathcal{E}}_{ia} &= \log\left(\frac{p_i(a)}{p_i(wt_i)}\right) + \sum_{j\in\mathcal{N}_i}\left\{\log\left(\frac{p_{ij}(a,wt_j)}{p_i(a)\,p_j(wt_j)}\right) - \log\left(\frac{p_{ij}(wt_i,wt_j)}{p_i(wt_i)\,p_j(wt_j)}\right)\right\} \\ &= (1-k_i)\big(\log p_i(a) - \log p_i(wt_i)\big) + \sum_{j\in\mathcal{N}_i}\Bigg(\log p_{ij}(a,wt_j) - \log p_{ij}(wt_i,wt_j)\Bigg) .\end{aligned} \tag{8}$$

Repeating the calculation above, we obtain the variance of this estimator:

$$\sigma_{ia}^2 \simeq \frac{1}{B}\left[\frac{|k_i-1|}{p_i(a)} + \frac{|k_i-1|}{p_i(wt_i)} + \sum_{j\in\mathcal{N}_i}\left(\frac{1}{p_{ij}(a,wt_j)} + \frac{1}{p_{ij}(wt_i,wt_j)}\right)\right] , \tag{9}$$

to the leading order in $1/B$. The presence of the absolute values $|\cdot|$ ensures the validity of the formula for non-interacting sites i, such that $k_i = 0$. Averaging Eq. (9) over the sites i and the mutations a yields Eq. 3 of the main text. Throughout this work, we considered only those mutations (i,a) that have at least one occurrence in the MSA to compute this average.

Appendix B: dependence of the squared bias on the mean Hamming distance to the wt sequence

Estimating the bias of a statistical model is generally complicated since it requires knowledge of the ground-truth probability distribution that generated the data. However, we show below that, under some simplifying hypothesis, the bias can be related to the mean Hamming distance between the sequences in the MSA and the wild-type sequence, wt.

This appendix is organized as follows. We first define the grounth-truth distribution of sequences, that is, the fitness landscape for sequences and estimate the effect of a mutation to wt. We then present the predictor of this mutational effect corresponding to the independent-site model, and derive a general formula for the bias that involves the amino-acid statistics in the MSA. We then estimate how these statistical quantities depends on the MSA properties in a simple probabilistic framework for generating MSA. Last of all, we discuss how these results are changed when the model used to predict mutational effects include epistatic couplings.

Change in log probability following a mutation

We assume that the protein family under consideration is defined by a distribution P over the space of sequences s. Informally speaking, good sequences s, *i.e.* corresponding to functional proteins have large values of P, while bad sequences correspond to very low values; We further assume that the landscape associated to P includes local biases acting on residues as well as pairwise epistatic interactions. More precisely, we have

$$P(s) = \frac{e^{-\mathcal{F}(s)}}{\mathcal{Z}} , \tag{10}$$

where $\mathcal{Z}$ is a normalization constant, and the "statistical energy" $\mathcal{F}(\mathbf{s})$ reads

$$\mathcal{F}(\mathbf{s}) = -\sum_i h_i(s_i) - \sum_{i<j} J_{ij}(s_i, s_j) \ . \tag{11}$$

According to Eq. (11), under the mutation $wt_i \to a$, the variation in the log probability of the sequence is equal to

$$\mathcal{E}_{ia} \equiv \log\left[\frac{P(wt \text{ with } wt_i \to a)}{P(wt)}\right] = h_i(a) - h_i(wt_i) + \sum_{j(\neq i)} \left[J_{ij}(a, wt_j) - J_{ij}(wt_i, wt_j)\right] \ . \tag{12}$$

In the following, $\mathcal{E}_{ia}$ defined in the above equation will be our ground-truth value for minus the fitness of the mutated sequence relative to wt: $\mathcal{E}_{ia} < 0$ corresponds to beneficial mutations, while $\mathcal{E}_{ia} > 0$ signals a deleterious mutation.

We remark that the distribution of the sequences in Eq. (10) is overparametrized. No change of the local biases of the form $h_i(s) \to h_i(s) + b_i$ will affect P, neither will changes to the couplings of the form $J_{ij}(s, s') \to J_{ij}(s, s') + c_{ij}(s) + d_{ij}(s')$. We may therefore, without any loss in generality, impose that all local fields and all couplings vanish when one of their amino acids coincide with the residue in the wt sequence, *i.e.* $h_i(wt_i) = J_{ij}(wt_i, s) = J_{ij}(s, wt_j) = 0$ for all residues s [3]. With this particular choice of gauge, hereafter referred to as wt gauge, the expression for the change in log probability following the mutation simplifies into

$$\mathcal{E}_{ia} = h'_i(a) \ , \tag{13}$$

where $'$ indicates the wt gauge.

Mean-field expression of bias for the independent-site model

Let us assume that we have generated a set of B sequences, called MSA, from the distribution defined in Eq. (10). We now want to build a model from this sequence data to have predictors of the mutational effects, $\widehat{\mathcal{E}}_{ia}$. In the simplest model, residues attached to different site are independent, and the probability of a sequence is simply given by

$$\widehat{P}(s) = \prod_i \frac{e^{\widehat{h}_i(s_i)}}{\widehat{\mathcal{Z}}_i} \ , \qquad \widehat{\mathcal{Z}}_i = \sum_s e^{\widehat{h}_i(s)} \ . \tag{14}$$

where the local fields (PWM) $\widehat{h}_i(s_i)$ are inferred to reproduce the statistics of residues in the MSA. Choosing again the wt gauge, that is, setting the field values for $s_i = wt_i$ to zero for all sites i, we have

$$\widehat{h}'_i(s_i) = \log p_i(s_i) - \log p_i(wt_i) \ , \tag{15}$$

where $p_i(s)$ is the frequency of amino acid s on site i in the data.

According to the independent-site model,the variation in the log probability of the sequence following the mutation $wt_i \to a$ is equal to

$$\widehat{\mathcal{E}}_{ia} \equiv \log\left[\frac{\widehat{P}(wt \text{ with } wt_i \to a)}{\widehat{P}(wt)}\right] = \widehat{h}'_i(a) \ , \tag{16}$$

where $'$ refers again to the wt gauge. The bias is therefore given by

$$\mu_{ia} \equiv \widehat{\mathcal{E}}_{ia} - \mathcal{E}_{ia} = \widehat{h}'_i(a) - h'_i(a) \ . \tag{17}$$

We now need to estimate this bias, more precisely, to compute $\widehat{h}'_i(a)$ from the ground-truth distribution in Eq. (10) defined by the parameters h_i, J_{ij}. To make this calculation tractable we resort to the so-called mean field approximation of statistical mechanics. According to mean-field theory the effective field $\widehat{h}'_i(s_i)$ acting on a site can be approximated as the sum of the local field, $h_i(s_i)$, and of the action of the other sites it is coupled to, substituted with their mean occupancies. More precisely,

$$\widehat{h}'_i(s_i) \simeq h'_i(s_i) + \sum_{j(\neq i)} \sum_{s_j} J'_{ij}(s_i, s_j)\, p_j(s_j) \ . \tag{18}$$

As a conclusion, based on Eq. (17), our mean-field expression for the bias in predicting the effect of mutation a on site i is equal to

$$\mu_{ia} = \sum_{j(\neq i)} \sum_{s_j} J'_{ij}(a, s_j)\, p_j(s_j) \ . \tag{19}$$

Two remarks are important here. First the bias vanishes when the couplings are equal to zero. Indeed, if the ground-truth distribution of amino acids factorizes over sites, then there is no systematic loss of accuracy in inferring the distribution with an independent-site model; statistical errors in inferring the fields from the MSA data will contribute to the variance term in the bias-variance trade-off, but the bias vanishes. Second, due to the choice of the wt gauge the coupling $J(a, s_j)$ vanish when $s_j = wt_j$. We may therefore rewrite the bias as

$$\mu_{ia} = \sum_{j(\neq i)} \sum_{s_j(\neq wt_j)} J'_{ij}(a, s_j)\, p_j(s_j) \ . \tag{20}$$

Relationship between the squared bias and the statistics of residues in the MSA

As our measure of goodness of prediction relies on the Spearman correlation between the experimental and predicted changes in fitness, respectively, ΔE_{ia} and $\widehat{\mathcal{E}}_{ia}$, it is left unchanged by any additive constant to $\widehat{\mathcal{E}}_{ia}$. We should therefore consider the centered bias

$$\mu_{ia} - \langle \mu_{ia} \rangle_{ia} = \sum_{j(\neq i)} \sum_{s_j(\neq wt_j)} \big(J'_{ij}(a, s_j) - \langle J'_{ij}(a, s_j) \rangle_{ia} \big)\, p_j(s_j) \ , \tag{21}$$

where $\langle \cdot \rangle_{ia}$ denotes the average over all sites i and residues a (different from the wt residue).

While we do not know the ground-truth fitness landscape from which sequences are drawn we may use DCA estimates of local fields and couplings as proxies for the true h_i and J_{ij}. In all cases studied in this work we find that the average coupling is very small compared to the standard deviation J_0, and can be replaced with zero. The centered bias is therefore practically equal to the bias.

We may now estimate the average squared bias over all sites and mutations

$$\mu^2 \equiv \langle \mu_{ia}^2 \rangle_{ia} = \sum_{j,k(\neq i)} \sum_{s_j, s_k(\neq wt_j)} \langle J'_{ij}(a, s_j)\, J'_{ik}(a, s_k) \rangle_{ia}\, p_j(s_j)\, p_k(s_k) \ . \tag{22}$$

Neglecting correlations between the couplings on different sites only pairs of identical sites $j = k$ carrying identical amino acids $s_i = s_k$ contribute to the sum above, with the result

$$\mu^2 = \big(1 - \frac{1}{N}\big)\, J^F\, Q \ , \tag{23}$$

where J^F is the variance of the couplings, N the length of the protein, and

$$Q \equiv \sum_j \sum_{s_j (\neq wt_j)} p_j(s_j)^2 \tag{24}$$

is a characterization of the MSA statistics. The validity of this formula is confirmed by studies of lattice-protein models, for which the bias can be calculated exactly, see Supplementary Fig. 1.

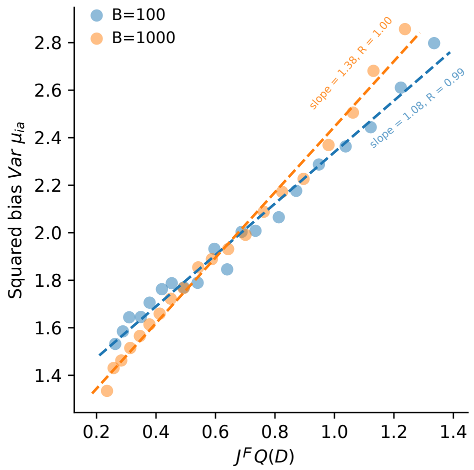

Supplementary Figure 1. Squared centered bias from Eq. (21) vs. $J^F \times Q$, where J^F is estimated as the variance of the couplings and Q is defined in Eq. (24), for the lattice-protein model associated to the structure shown in Main text Fig. 3A.

We show in the next subsections that the average squared frequency Q is, to a very good approximation, proportional to the mean Hamming distance of the sequences in the MSA to the wt. This result justifies the linear dependence of the squared bias μ^2 upon D reported in the main text.

Statistical properties of the subsampled MSA

Consider a multi-sequence alignement (MSA) with B_{tot} sequences of length N. For the sake of mathematical tractability, we assume that all sequences are drawn from a background (bg) distribution, where the probability of the amino acid a is denoted by $bg(a)$, see for instance Carugo, O. (2008), Amino acid composition and protein dimension. Protein Science, 17: 2187-2191. https://doi.org/10.1110/ps.037762.108. We now build a sub-MSA as follows:

- One of the sequences is the MSA is called wt and included in the sub MSA;
- Each other sequence in the MSA is retained with probability $\propto e^{-\alpha\, d}$, where d is its Hamming distance to wt, and α is a positive parameter.

We want to estimate, as functions of α: (1) the mean Hamming distance of the sequences in the sub-MSA to wt, $D_{sub-MSA}$; (2) the mean number of sequences in the sub-MSA, $B_{sub-MSA}$; (3) the average squared frequencies, $Q_{sub-MSA}$, entering eqn (24).

We first focus on all sequences in the sub-MSA but wt. According to the sub-sampling procedure above, the probability of amino acid a on site i is proportional

to $bg(a)$ if $a = wt_i$ and to $e^{-\alpha}\, bg(a)$ if $a \neq wt_i$. The mean Hamming distance of sequences to wt is therefore

$$D(\alpha) = \sum_{i=1}^{N} \left(\frac{e^{-\alpha}\,(1 - bg(wt_i))}{bg(wt_i) + e^{-\alpha}\,(1 - bg(wt_i))} \right) = N \sum_a \frac{bg(a)(1 - bg(a))\, e^{-\alpha}}{bg(a) + (1 - bg(a))\, e^{-\alpha}} \tag{25}$$

where the last equality comes from the average over the wt sequence. Similarly, we get

$$Q(\alpha) = \sum_{i=1}^{N} \sum_{a'(\neq wt_i)} \left(\frac{e^{-\alpha}\, bg(a')}{bg(wt_i) + e^{-\alpha}\,(1 - bg(wt_i))} \right)^2 = N \sum_a \frac{bg(a)\,(bg^{(2)} - bg(a)^2)\, e^{-2\alpha}}{\big(bg(a) + e^{-\alpha}\,(1 - bg(a))\big)^2} \,, \tag{26}$$

where, for integer-valued k,

$$bg^{(k)} = \sum_a bg(a)^k \ . \tag{27}$$

To estimate the number of sequences that are subsampled, we consider the generating function of distances,

$$\mathcal{G}(\alpha; wt) = \prod_{i=1}^{N} \big(bg(wt_i) + e^{-\alpha}(1 - bg(wt_i))\big) = \sum_{d=0}^{N} \mathcal{P}(d)\, e^{-\alpha\, d} \ , \tag{28}$$

where $\mathcal{P}(d)$ is the probability that a sequence is at distance d from wt. The average number of sub-sampled sequences can therefore be approximated as

$$B(\alpha) \simeq (B_{tot} - 1) \times \mathcal{P}\big(D(\alpha)\big) \simeq (B_{tot} - 1) \times \mathcal{G}(\alpha; wt) \times e^{+\alpha\, D(\alpha)} \simeq (B_{tot} - 1) \times e^{N\, \omega(\alpha)} \,, \tag{29}$$

where

$$\omega(\alpha) = \sum_a bg(a) \left\{ \ln \big(bg(a) + e^{-\alpha}(1 - bg(a))\big) + \frac{\alpha\,(1 - bg(a))\, e^{-\alpha}}{bg(a) + (1 - bg(a))\, e^{-\alpha}} \right\} \tag{30}$$

after averaging of $\log \mathcal{G}$ over the wt sequence.

Inserting back the wt sequence in the sub-MSA, we obtain the following expressions for the three quantities of interest:

$$B_{sub-MSA} = B(\alpha) + 1 \ , \tag{31}$$

$$D_{sub-MSA} = \frac{B(\alpha)}{B(\alpha) + 1}\, D(\alpha) \ , \tag{32}$$

$$Q_{sub-MSA} = \frac{B(\alpha)}{B(\alpha) + 1}\, Q(\alpha) \ . \tag{33}$$

Approximate linear dependence of Q upon D

We now parametrically plot the number $B_{sub-MSA}$ of sequences in the sub-MSA and the averaged squared frequencies $Q_{sub-MSA}$ vs. the average Hamming distance $D_{sub-MSA}$ by varying α from 0 (all B_{tot} sequences are considered) to $+\infty$ (all sequences are left out, with the exception of wt. Results are shown in Supplementary Fig. 2. We observe that Q is approximately a linear function of the distance D over a large range of sub-sampling levels of the full MSA.

An estimate of the slope β can be computed through an expansion of $\omega(\alpha)$ in eqn (30) to the second order in α. We obtain

$$\omega(\alpha) \simeq -\frac{\alpha^2}{2}\big(bg^{(2)} - bg^{(3)}\big) \ . \tag{34}$$

Based on this quadratic approximation, we conclude:

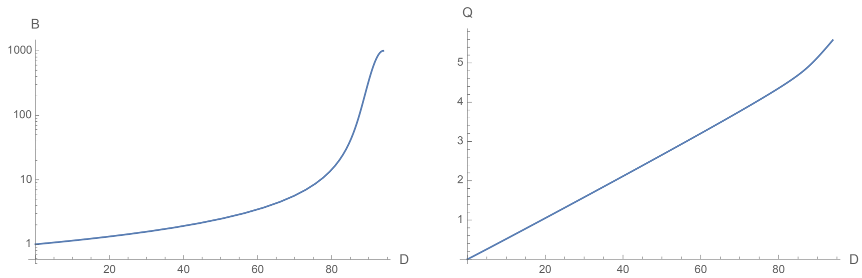

Supplementary Figure 2. Number $B_{sub-MSA}$ of sequences in the sub-MSA (left) and averaged squared frequencies $Q_{sub-MSA}$ (right) vs. average Hamming distance $D_{sub-MSA}$, obtained from eqns. (31), by subsampling a MSA with $B_{tot} = 1000$ sequences of length $N = 100$. The approximate linear scaling of Q with D is valid even when the full MSA is very strongly subsampled. The parts of the curves corresponding to distances $D \leq D_{min} \simeq 40$ include on average less than 2 sequences in the sub-MSA (hence a random realization of the sub-MSA may contain the *wt* only), and should be discarded, see text.

- For $\alpha = 0$, the full MSA is retained through sub-sampling, with

$$Q_{sub-MSA} \simeq Q(0) = N\left(bg^{(2)} - bg^{(3)}\right) . \tag{35}$$

  The average distance to *wt* is

$$D_{sub-MSA} \simeq D(0) = N\left(1 - bg^{(2)}\right) . \tag{36}$$

- As α increases, the sub-MSA shrinks until it contains *wt* only. This happens for $\alpha = \alpha_{min}$ such that

$$B(\alpha_{min}) = 1 \implies \alpha_{min} \simeq \sqrt{\frac{\ln B_{tot}}{N\left(bg^{(2)} - bg^{(3)}\right)}} . \tag{37}$$

  We see that α_{min} is small, and the quadratic expansion is accurate for long sequences or small MSA.

- For larger values of α, the predictions of eqn (31) are meaningless since they are based on sub-MSA with, on average, less than 2 sequences, see Supplementary Fig. 2. In practice, most sub-MSA contain *wt* only, and $Q_{sub-MSA} = 0$.

We can therefore estimate the slope through

$$\beta \equiv \frac{dQ}{dD} \simeq \frac{Q(0)}{D(0)} \simeq \frac{bg^{(2)} - bg^{(3)}}{1 - bg^{(2)}} . \tag{38}$$

The above expressions were obtained with a uniform background model along the sequence, but can easily be extended to the case of $bg(a)$ varying from site to site.

Case of non-homogeneous pairwise couplings: inference with the sparse-Potts model

Formula (23) above implicitly assumes that all couplings $J_{ij}(a, b)$ have the same variance J_0,independently of the sites i, j under consideration. In reality, however, the couplings entering the 'ground-truth' statistical energy $\mathcal{F}(s)$ (11) may considerably vary with the pairs of sites i, j. This situation may in particular arise when the sites i, j are in contact on the protein fold.

For the sake of simplicity, let us assume that

- there are M_+ pairs of sites i, j such that the variance of the couplings (computed over the amino acids carried by the sites) is

$$\langle J'_{ij}(a,b)^2 \rangle_{a,b} = J^F_+ \ . \tag{39}$$

- for the remaining $M_- = \frac{1}{2}N(N-1) - M_+$ pairs of sites i, j the variance of the couplings is

$$\langle J'_{ij}(a,b)^2 \rangle_{a,b} = J^F_- \ll J^F_+ \ . \tag{40}$$

Assume we now predict the mutational effect with the sparse Potts model, with K couplings allowed to take non-zero values. Equation (10) for the probability of a sequence is therefore substituted with

$$\widehat{P}(s) = \frac{e^{-\widehat{\mathcal{F}}(s)}}{\widehat{\mathcal{Z}}_i} \qquad \text{where} \qquad \widehat{\mathcal{F}}(\mathbf{s}) = -\sum_i \widehat{h}_i(s_i) - \sum_{i<j} \widehat{J}_{ij}(s_i, s_j) \tag{41}$$

and $\widehat{\mathcal{Z}}$ is a normalizing factor.

The calculation of the predicted change in log probability following a mutation is straightforward from the previous considerations, and eqn (17) for the bias is still valid. However, upon application of mean-field theory to both the inferred sparse Potts model and the ground-truth distribution, eqn (18) becomes

$$\widehat{h}'_i(s_i) + \sum_{j(\neq i)} \sum_{s_j} \widehat{J}'_{ij}(s_i, s_j)\, p_j(s_j) \simeq h'_i(s_i) + \sum_{j(\neq i)} \sum_{s_j} J'_{ij}(s_i, s_j)\, p_j(s_j) \ . \tag{42}$$

We may conclude that the bias is now equal to

$$\mu_{ia} = \widehat{h}'_i(a) - h'_i(a) = \sum_{j(\neq i)} \sum_{s_j(\neq wt_j)} \left(J'_{ij}(a, s_j) - \widehat{J}'_{ij}(a, s_j) \right) p_j(s_j) \ . \tag{43}$$

Case $K \leq M_+$

If the Potts model used to reproduce the fitness landscape is very sparse, the K inferred couplings $\widehat{J}'$ are likely to be good approximations of the K largest ground-truth epistatic couplings J' (having variance J^F_+). As a consequence, K terms in the sum in eqn (43) vanish. After averaging over the sites and amino acids, the squared bias μ^2 is given by

$$\mu^2 = \left(1 - \frac{1}{N} \right) J^F(K)\, Q \ , \tag{44}$$

where Q is given by eqn (24), and

$$J^F(K) = \frac{(M_+ - K) J^F_+ + M_-\, J^F_-}{\frac{1}{2}N(N-1) - K} \ . \tag{45}$$

Notice that the effective coupling variance $J^F(K)$ is a decreasing function of K: as more and more couplings are included in the inferred Potts model less and less epistatic effects remain un-modelled and contribute to the bias.

Case $K > M_+$

Equation (44) still holds, with

$$J^F(K) = J^F_- \ . \tag{46}$$

In this regime, the effective coupling variance does not vary with K any longer.

References

1. Barton JP, De Leonardis E, Coucke A, Cocco S. ACE: adaptive cluster expansion for maximum entropy graphical model inference. Bioinformatics. 2016;32(20):3089–3097.

2. Cocco S, Posani L, Monasson R. Functional effects of mutations in proteins can be predicted and interpreted through guided selection of sequence covariation information. Submitted for publication. 2023;.

3. Cocco S, Feinauer C, Figliuzzi M, Monasson R, Weigt M. Inverse statistical physics of protein sequences: a key issues review. Reports on Progress in Physics. 2018;81(3):032601.
