# Supplementary figures and images for: Infer global, predict local: Quantity-relevance trade-off in protein fitness predictions from sequence data
Source: PLoS Comput Biol. 2023 Oct 26;19(10):e1011521. doi: 10.1371/journal.pcbi.1011521 (PMC10645369; doi:10.1371/journal.pcbi.1011521)

BLAT

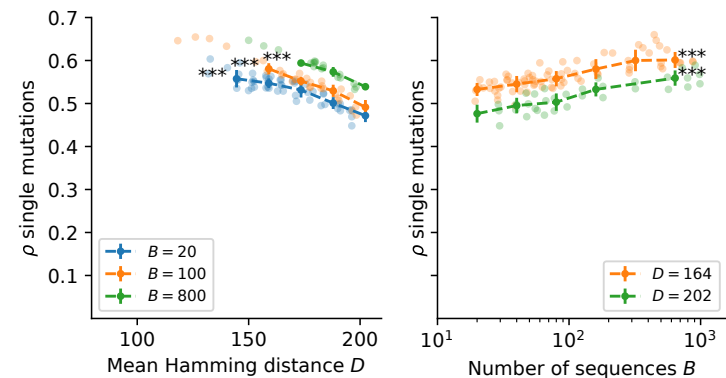

DNA-BIND

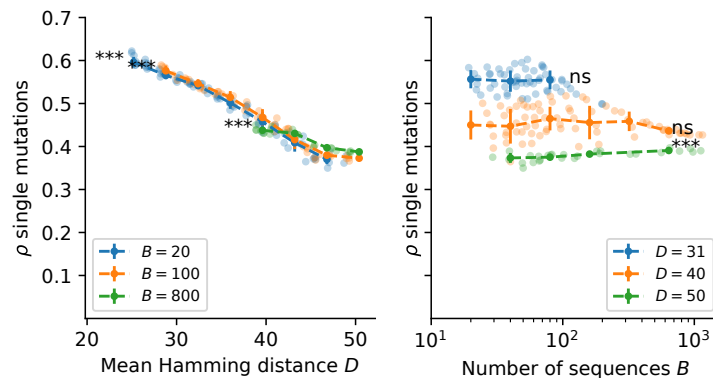

RL401

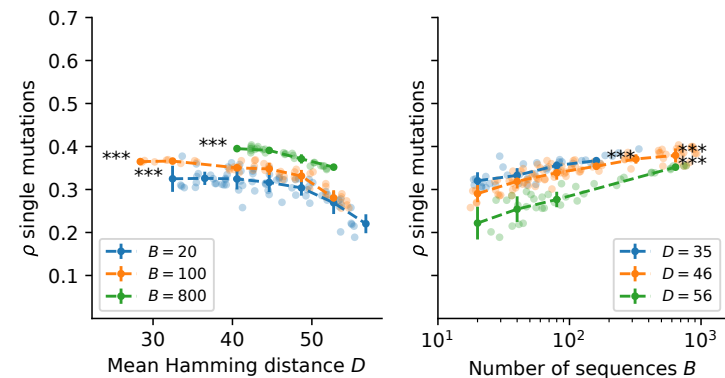

PDZ

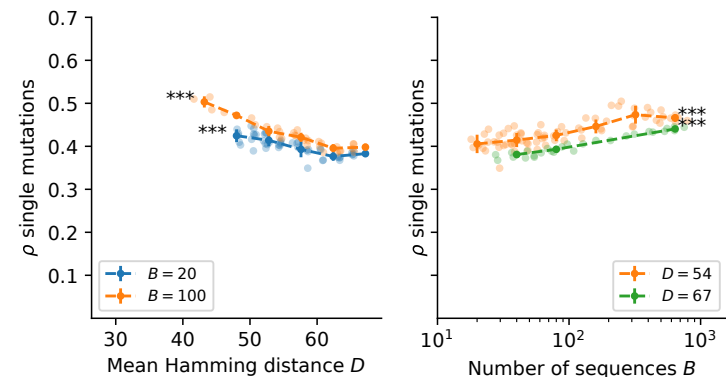

UBOX

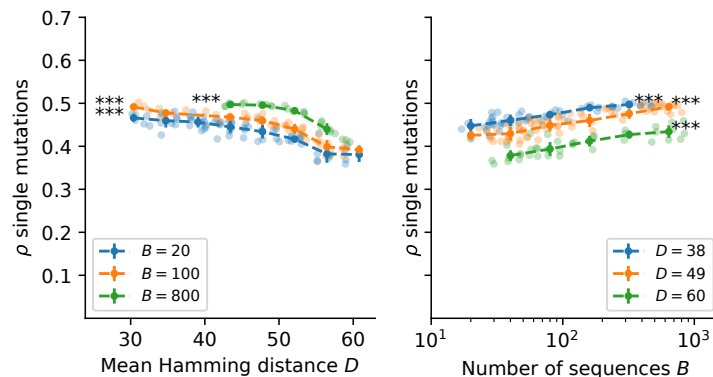

WW

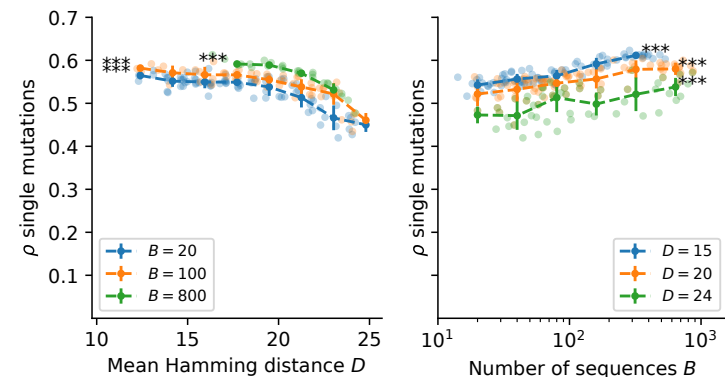

Supplement: S1 Fig — Same as Main text Fig 2D for all protein families except RNA-Bind (shown in Main text Fig 2D): systematic analysis of the predictive power ρ as a function of the mean Hamming distance D of sub-alignments with fixed size B (left panels), and of the sub-alignment size B at fixed Hamming distance D (right panels). Each point represents the binned average and standard deviation of several sub-samples obtained at the corresponding values of D and B (see Methods). All significance levels refer to Spearman rank correlation. * P < 0.05; ** P < 0.01; *** P < 0.001. (PDF) [file pcbi.1011521.s002.pdf]

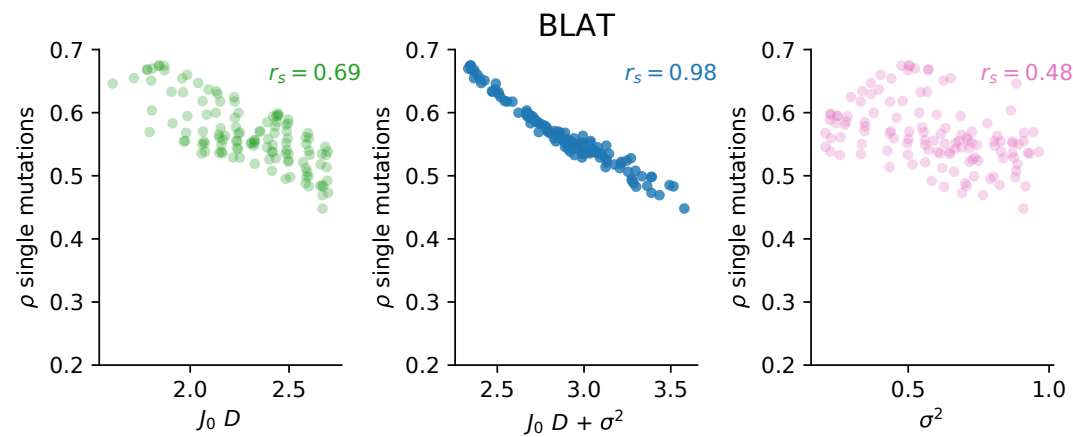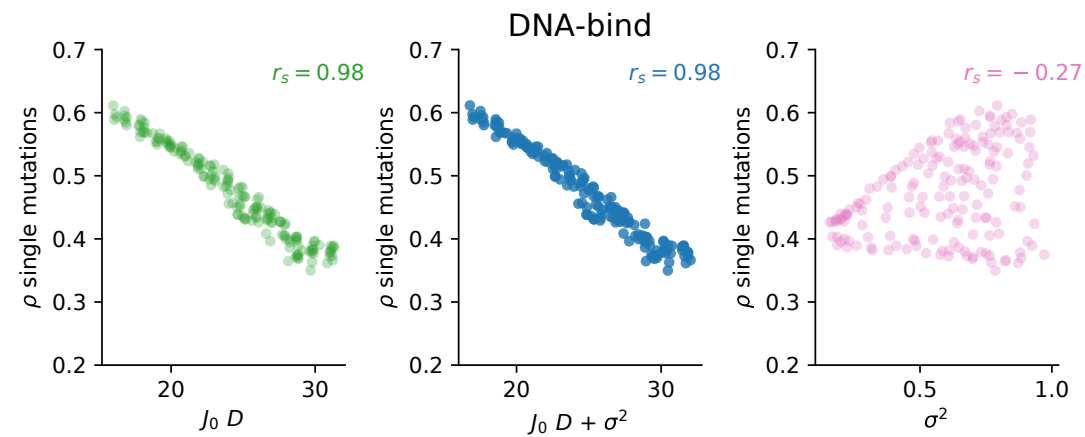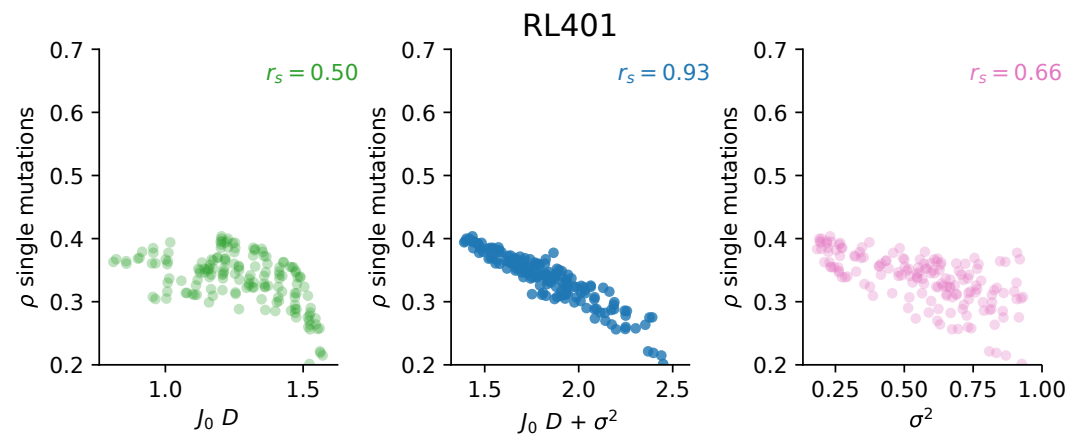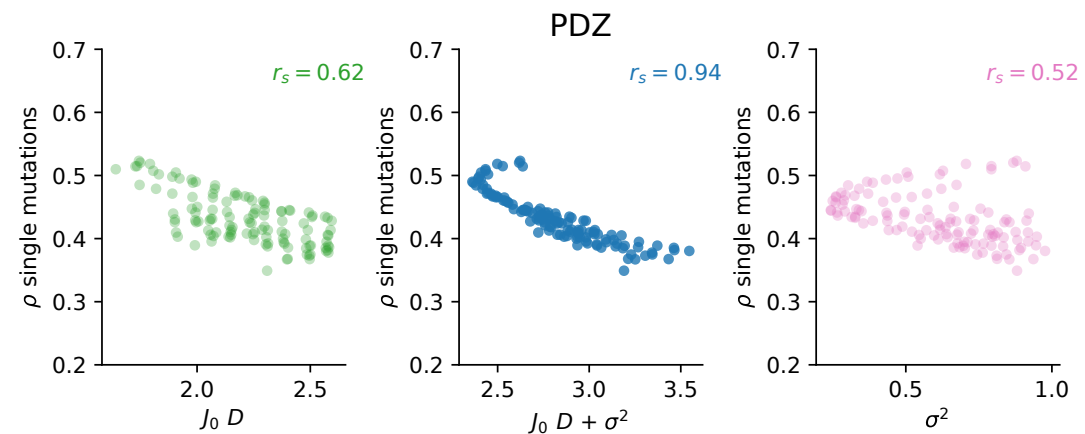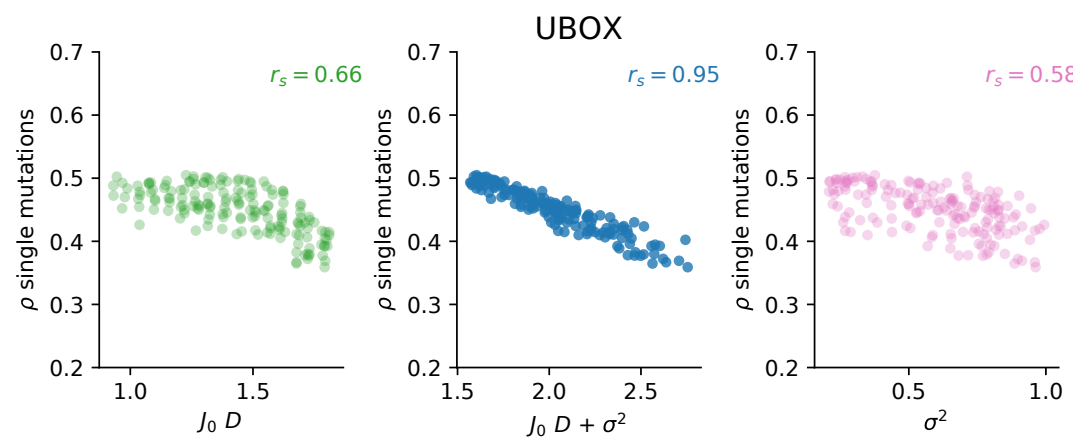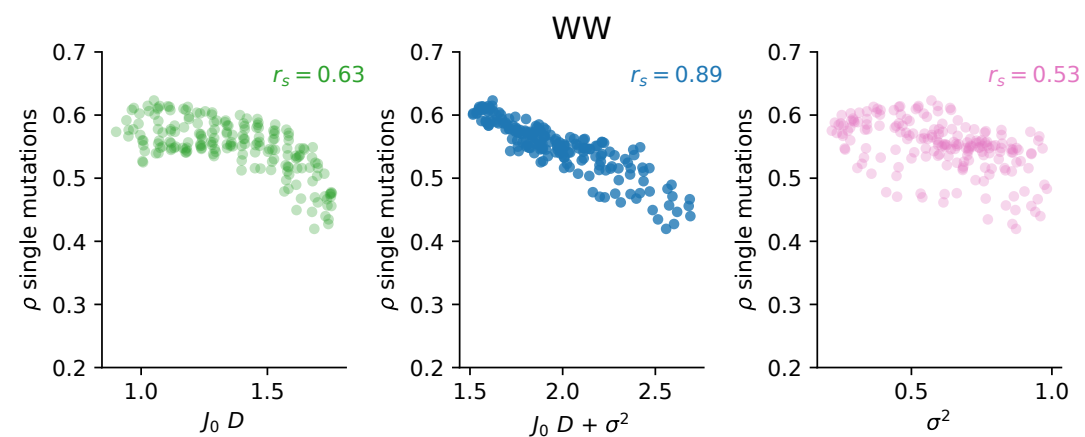

Supplement: S2 Fig — Same as Main text Fig 4A&4B for all protein families except RNA-Bind (shown in Main text Fig 4A&4B): predictive performance of single-point mutations using the independent-site models, as a function of the squared bias and variance estimated from the alignments, separately (left and right panels) and combined (central panel). (PDF) [file pcbi.1011521.s003.pdf]

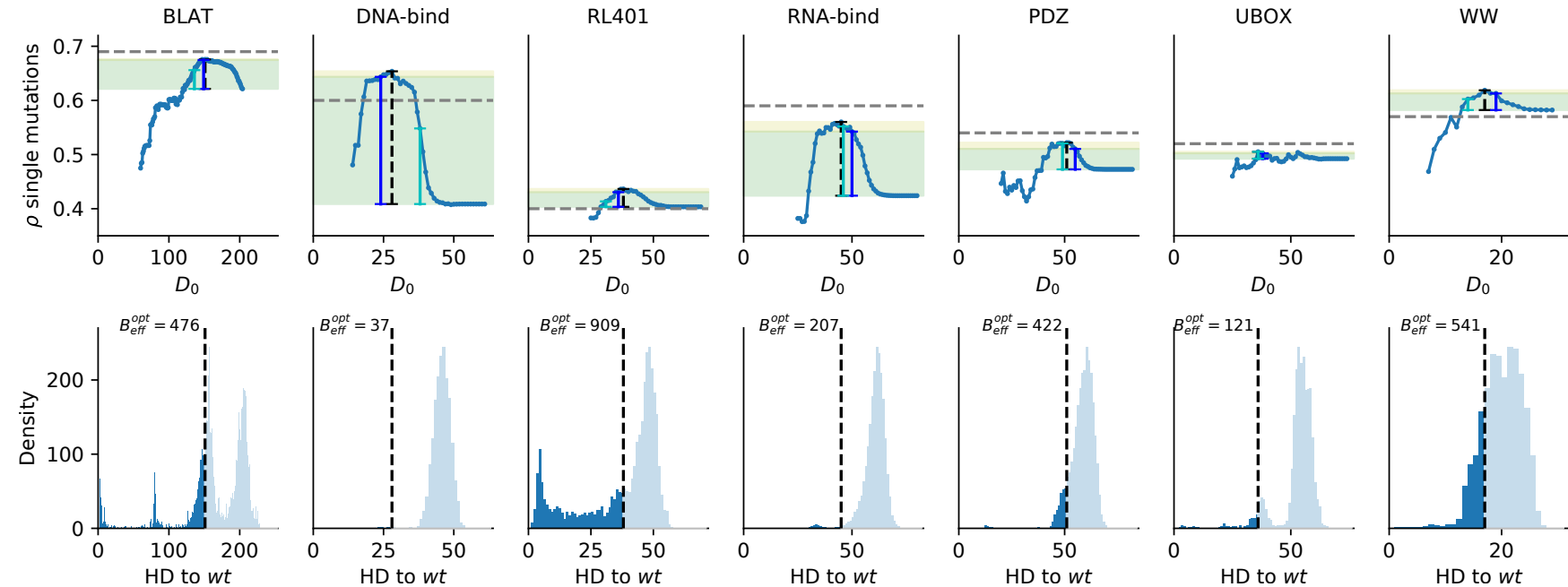

Supplement: S3 Fig — Top: single mutation prediction performance of the independent Potts model (K = 0) along the focusing axis (as a function of the cutoff distance D0) for the 7 studied protein families. Black dashed lines indicate the optimal cutoffs dopt; blue lines indicate the predicted cutoffs dbv by minimizing the linear sum of bias and variance; the light blue lines indicate the predicted cutoff from the signal-to-noise heuristic dsnr. Green areas highlight the performance increase from the full alignment (dc = N) to the predicted cutoff dbv. Yellow areas indicate the remaining performance increase to the optimal cutoff dopt. Horizontal dashed grey lines indicate the performance reported in [37] with a fully connected Potts model inferred by pseudo-likelihood. Bottom: distribution of the hamming distance to the wildtype D of sequences in the MSA. Black dashed lines indicate the optimal cutoff at which the best performance is reached. Beffopt is the effective number of sequences remaining in the MSA at the optimal cutoff. Refer to Table 1 in Methods for the original number of sequences in the MSA. (PDF) [file pcbi.1011521.s004.pdf]

**a**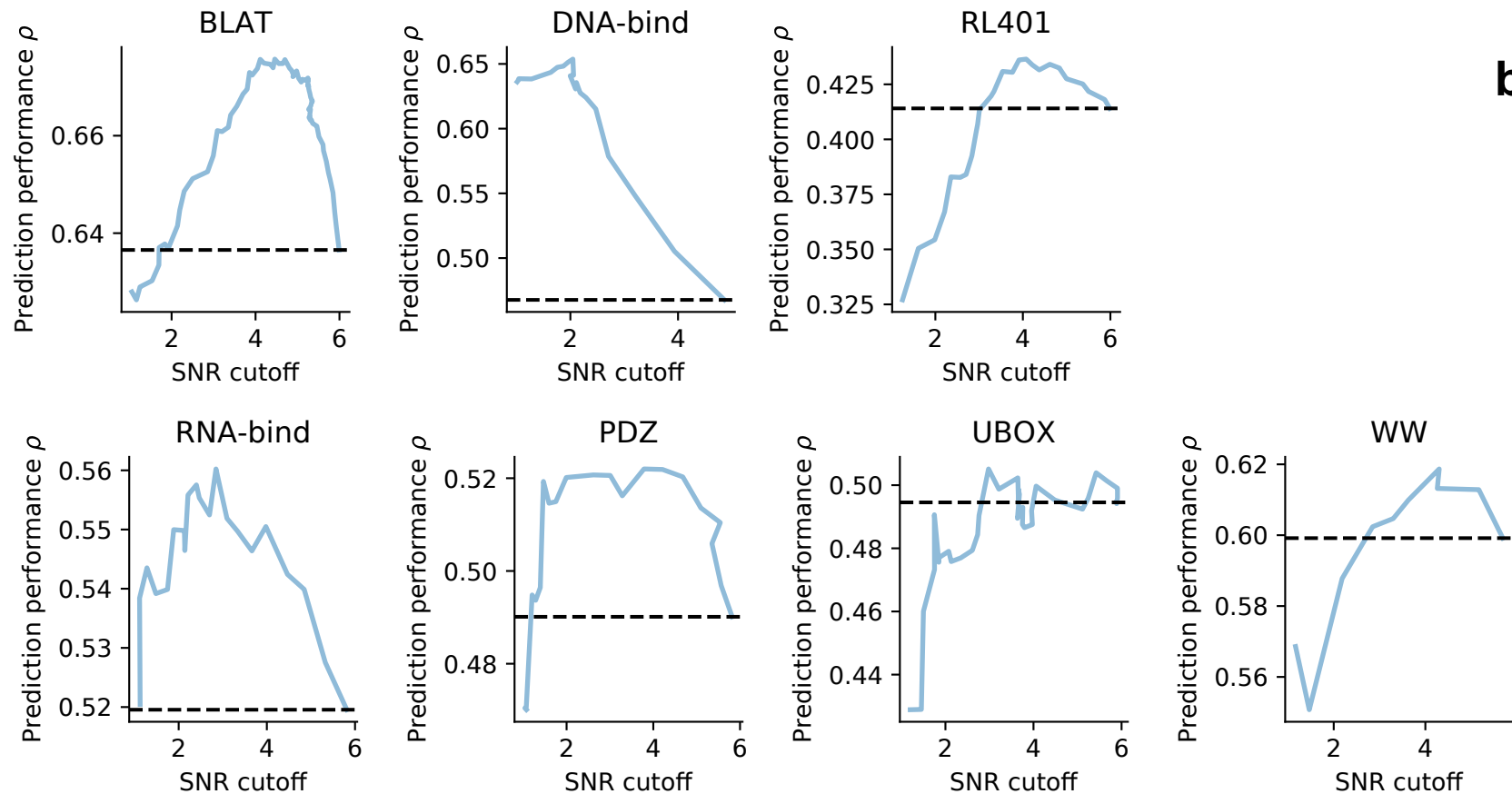**b**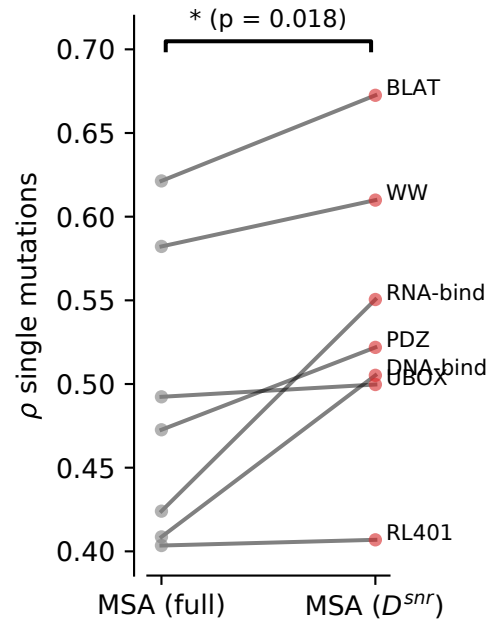

Supplement: S4 Fig — a: Single mutation prediction performance of the independent model at the predicted optimal cutoff using the SNR method, as a function of the SNR threshold, for the 7 protein families. b comparison between performance without any cutoff (MSA full) and performance at the cutoff predicted by using the rule of thumb SNR = 3. (PDF) [file pcbi.1011521.s005.pdf]

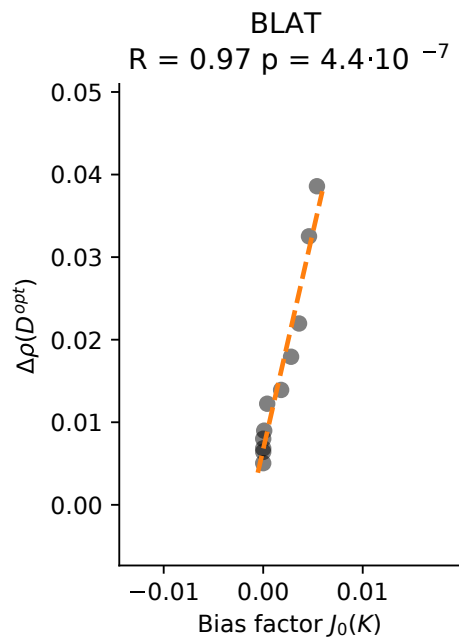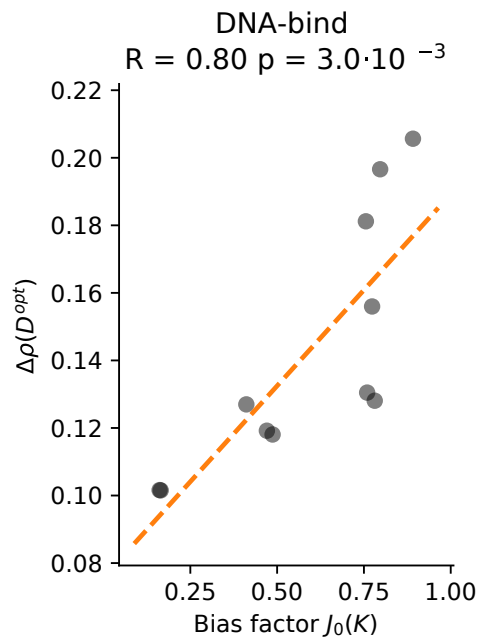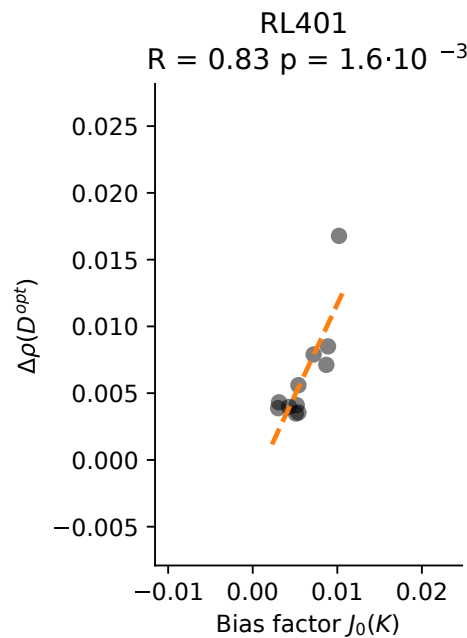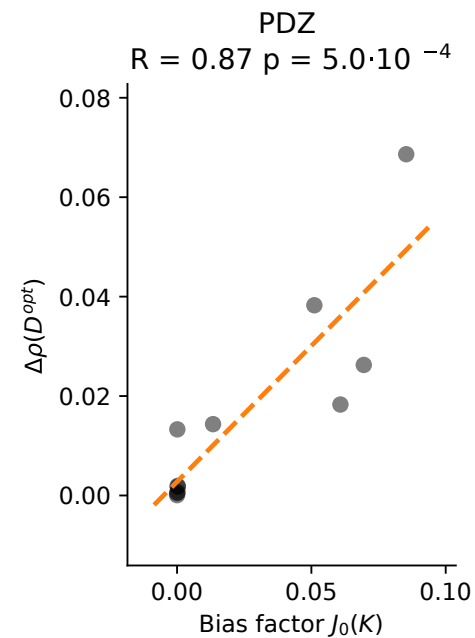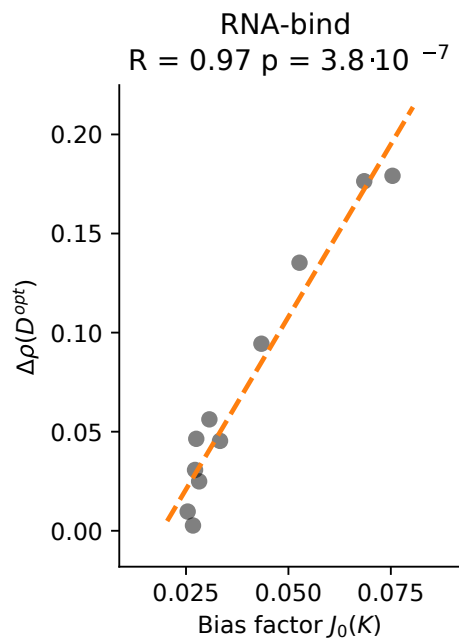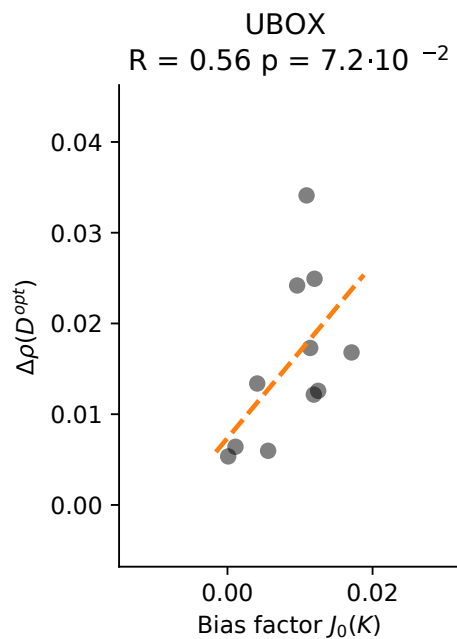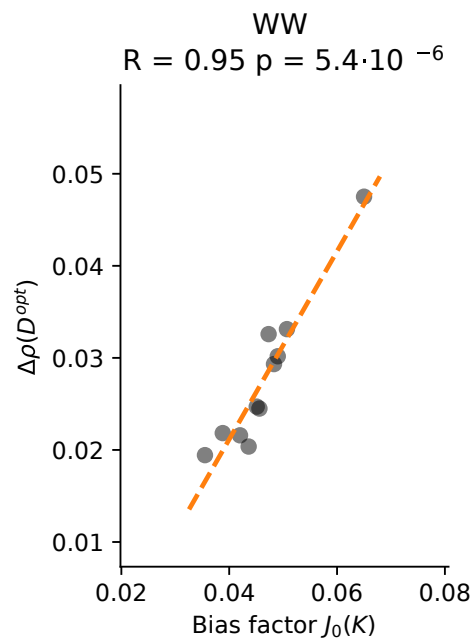

Supplement: S5 Fig — Relation between the bias factor J0(K) and improvement Δρ(dopt) for the optimal focusing cutoff for the 7 studied protein families. For each family, K is varied between 0 and N (number of sites in the alignment). (PDF) [file pcbi.1011521.s006.pdf]
