# Supplementary material for: Infer global, predict local: Quantity-relevance trade-off in protein fitness predictions from sequence data
Source: PLoS Comput Biol. 2023 Oct 26;19(10):e1011521. doi: 10.1371/journal.pcbi.1011521 (PMC10645369; doi:10.1371/journal.pcbi.1011521)
Supplement: Attachment — Submitted filename: infer_global_predict_local_review.pdf [file pcbi.1011521.s007.pdf]

The authors explore the question: when using a particular model for a prediction task, how should one optimally pick the training data? There is a tradeoff between the “quality” (really, how relevant is the data? see comment 3) and the “quantity” of the data. The authors consider the task of approximating the fitness of sequences around a given wild type sequence using Potts models trained on MSAs. In short, they find that it is possible to improve predictive performance by subsampling the MSA to bias it towards sequences that are sufficiently similar to the wild-type. Much of the paper is focused on empirically and analytically deriving how to subsample to optimize performance. The topic is interesting, well-motivated, and I believe to be of interest to the PLOS Computational Biology readership.

Two strengths of the paper are (i) the discussion of the parallel between the quality-quantity tradeoff described here and the well-known bias-variance tradeoff in the statistics literature, and (ii) the synthesis of analytic derivations, experiments on synthetic data where the “true” fitness function is known, and applications on seven real different datasets. My two major comments are as follows:

1. The authors claim that the error due to bias “reveals how much of the epistatic interactions shaping the fitness landscape are left unmodeled.” I am not convinced that this is true in general. In particular, they give an example (lines 190-194):

   *Assume first that we use the independent-site model for fitness prediction. If the ‘true’ fitness landscape shows no epistasis, this model is exact (up to statistical fluctuations due to the finite amount of training data, taken care of by σ2), and the bias vanishes. Therefore, a non zero bias would signal the presence of epistatic interactions between residues not captured by the simple model used for predictions.*

   I do not follow this logic. With a ‘true’ linear fitness landscape, bias will occur if the empirical frequencies of the residues at a position are not perfectly correlated with the additive fitness effects of the corresponding substitutions at that position. To be concrete, imagine at a fixed position the fitness effect of amino acid A is greater than the fitness effect of amino acid B, but in the MSA B is more common than A. The model, which is based on the assumption that empirical frequencies correlate with the fitness effect, will infer that B is more fit than A, incurring an error that has nothing to do with epistasis. (This type of scenario could occur for many reasons: perhaps A confers some disadvantage in translation in some organisms, perhaps A is more present across the whole protein family but the full MSA we have is a biased subsample based on what we have sequenced where B happens to be more common, perhaps there is a population genetic reason for the relative abundance of B.)

   Figure 5C shows a trend that as the model includes more epistatic terms (K), the bias decreases. This suggests that in these examples the bias (in particular for low K) might be due to unmodeled epistatic interactions. Nonetheless, I don’t think it is fair to in general equate bias, which could be caused by a number of forms of model misspecification, with unmodeled epistasis.

2. The authors propose an empirical rule for defining a cutoff (d_snr) to determine which sequences to include in the MSA for fitness effect prediction with a Potts model. I suggest that the authors more cohesively present the predictive performance of the model when d_snr is used. To this end (i) please address how the SNR threshold of 3 was chosen (presumably fit on these 7 families; perhaps better to fit on a subset of the families and evaluate on the other families; having only 7 families makes this difficult), (ii) produce the analog of Figure 4E for the d_snr cutoffs and include it in Figure 4 (it is hard to deduce this from Fig 4F), and (iii) reference Supplementary Figure 5 in the section "Cutoff for optimal focusing can be reliably predicted from heuristics" (Line 324).

Additional comments:

3. The choice of "quality" to describe which sequences are best to use for a specific task is a bit odd. Generally, one thinks of a "quality" MSA as one containing a diverse set of well-aligned sequences. In the paper, a high-quality MSA refers to one where the degree of similarity between the sequences and a given wild type is optimal for some downstream task. Instead of describing the tradeoff as "quality" versus "quantity", perhaps it is better to frame it as "relevance" versus "quantity". It is not that the MSA becomes higher quality when you remove dissimilar sequences, but rather the sequences that remain are the most relevant for the prediction task.
4. Equation 1, which expresses the mean squared error as the sum of bias and variance, is difficult to parse due to notation. The authors use *E-hat-i-a* to express both the (i) the predicted fitness when amino acid *a* is at position *i* and (ii) the expectation of this quantity under repeated predictions with different subsets of data (with same quality and quantity statistics). A literal reader would conclude that the variance is always zero. Please improve the notation.
5. Please add a couple sentences about lattice proteins (e.g. how are the folds determined) to the main text to make the section more self-contained. (Line 209)
6. The subsection "J_0 can be inferred without any knowledge of the true fitness landscape" (Line 265) describes how to estimate J_0 using rho, the predictive performance. Computing rho requires the measured fitness, which is something about the fitness landscape. The title of the subsection does not make sense.
7. Fig 4E + Lines 313-323: To what extent is the improvement in performance between MSA(full) and MSA(d_opt) correlated with the difference in average Hamming distance to the wt in MSA(full) and MSA(d_opt)? (Sup Fig 5 suggests that it is.)
8. Fig 5C: how might you account for the non-monotonicity in the curves (in particular WW)?
9. Appendix A: Please justify the approximation <p>(1-<p>) = <p>. Please explain where the (1-k_i) term in equation (20) comes from. How does the reweighting procedure affect the estimate (21)? Are the p_i(a) computed with respect to the weights on the sequences within the MSA?
10. AlphaFold predictions are known to be sensitive to the input MSA; in CASP15 some methods found improved predictions by changing the way MSAs were generated (see

e.g. Elofsson, 2023, *Progress at protein structure prediction, as seen in CASP15*). Perhaps this is worth mentioning to further your point that the selection of sequences is an important part of a task.

11. What value of K was used in producing Supplementary Fig 5? Please include in the caption.
12. I could not find code to reproduce the results in the paper.

Typos/style:

1. The caption of Fig 1 refers to a grey dashed line, but there is no such line in the figure.
2. Line 128 and caption of Fig 2: wrong character for <
3. Don’t start sentences with mathematical characters (e.g. Lines 181, 205)
4. Mismatched quotes; use `` instead of “ for left quotes in LaTex (e.g. Line 307, 477)
5. and We → , and we (Line 351)
6. decrease → decreases (Line 367)
7. Fig 4A: add label to y-axis
8. Equations 13 and 14: some rendering issues? The = missing?
9. grounth → ground (Line 763)
