# Supplementary material for: Infer global, predict local: Quantity-relevance trade-off in protein fitness predictions from sequence data
Source: PLoS Comput Biol. 2023 Oct 26;19(10):e1011521. doi: 10.1371/journal.pcbi.1011521 (PMC10645369; doi:10.1371/journal.pcbi.1011521)
Supplement: Attachment — Submitted filename: Answers_Revisions PLOS CB.pdf [file pcbi.1011521.s008.pdf]

Answer to the Referees

We thank the Referees for their positive comments on our manuscript. We reproduce below their remarks (in black), as well as our detailed answer (in blue).

Reviewer 1

This is a well-written paper on the important topic of deciding which kind of data (large but of worse quality or of more moderate size but of better quality) is more appropriate to infer fitness landscapes. The inference procedure considered in the paper is model learning in exponential families (or Direct Coupling Analysis). As is well known today this is inferior to deep learning / AlphaFold / on the flagship application of predicting protein structure, but still a method of choice when large training data is not available. Also, this kind of inference procedure is, though also somewhat complex, still sufficiently simple that it may be possible to analyse performance ab initio.

Several kinds of data are considered in the paper, and are clearly described. The results are also presented clearly. A strong aspect of the paper is the extensive use of data from deep mutational scans.

I hence recommend acceptance, provided some minor adjustments are made.

1. The authors focus on a mean-square characterization of errors, but as the authors surely know this is not the only possibility. It is also not the main characterization of performance of this class of methods on real data; instead a version of "top-k" is more commonly adopted. This importance of inference criteria in this context has been stressed e.g. by Aurell and co-workers (most recently in arXiv:2205.00750), but other points of view can also be found in the literature.

Important technical contributions include:

- *M. Vuffray, S. Misra, A. Lokhov, and M. Chertkov. Interaction screening: Efficient and sample-optimal learning of Ising models.*
- *In D. D. Lee, M. Sugiyama, U. V. Luxburg, I. Guyon, and R. Garnett, editors, Advances in Neural Information Processing Systems 29, pages 2595–2603. Curran Associates, Inc., 2016.*
- *A. Y. Lokhov, M. Vuffray, S. Misra, and M. Chertkov. Optimal structure and parameter learning of Ising models. Sci. Adv., 4(3):e1700791, March 2018.*
- *J. Berg. Statistical mechanics of the inverse Ising problem and the optimal objective function. J. Stat. Mech: Theory Exp., 2017(8):083402, 2017.*

mostly for other variants of DCA than considered by the authors, but also including pseudo-likelihood maximization. The gist of these contributions is that if the goal is "top-k" (as it

has often been in practice), then very few samples are often enough to achieve good performance.
I ask the authors to include in a revised version of their paper a short section where they conceptualize this issue and discuss various pros and cons.

We agree with Reviewer 1 that top-k selection is particularly appropriate for contact predictions, which was the original scope of DCA. In this context, it is crucial to estimate the fraction of structural contacts among the k largest inferred couplings. The inference problem then boils down to the identification of the support of the interaction network, a task which can be done with relatively few samples (typically, of the order of the logarithm of the system size) with methods such as plmDCA or the ones cited by Reviewer 1.

In the present work, we focus on mutation effects predictions, and the Spearman correlation between single-point mutations and their estimators is a more adequate and standard measure of the goodness of the model. From a theoretical point of view, the choice of the mean-squared error is made natural by the well-known decomposition into (squared) bias and variance. Both terms can be analytically computed (to some degree of approximation). In addition, we empirically show in our manuscript through data analysis that the Spearman correlation is monotonically related to the sum of the squared bias and of the variance, which ultimately justifies the use of this quantity.

We have added further explanations about the choice of the mean-squared error in lines 153-157.

2. Two relevant theoretical papers which also deserve to be cited and briefly commented are

- *L. Bachschmid-Romano and M. Opper. A statistical physics approach to learning curves for the inverse ising problem. Journal of Statistical Mechanics: Theory and Experiment, 2017(6):063406, jun 2017.*
- *A. Abbara, Y. Kabashima, T. Obuchi, and Y. Xu. Learning performance in inverse ising problems with sparse teacher couplings. Journal of Statistical Mechanics: Theory and Experiment, 2020(7):073402, jul 2020.*

3. The authors are encouraged to survey the theoretical literature to make sure that later paper in the direction of the above are not missed. It is important for coherence of the field that contacts between theory-driven and data-driven contributions are maintained.

Reviewer 1 has a valuable point in stressing that theory- and data-driven developments in the field of graphical models should not depart from one another. We have added a comment and citations to these papers in the Discussion section (lines 560-573) to emphasize the similarities and differences between them and our approach (beyond the obvious fact that our paper is strongly motivated by protein studies).

In particular, while the papers above rely on a statistical consistency (also called teacher-student) framework, our approach acknowledges the fact that models are generally simpler than reality. In fact, we mostly use independent-site models for inference in our manuscript. Our goal is to provide some theoretical understanding of why adapting the data to the models can help in many situations, as empirically known in various protein-related contexts (see Answer to Reviewer 2).

Reviewer 2

The topic is interesting, well-motivated, and I believe to be of interest to the PLOS Computational Biology readership.
Two strengths of the paper are (i) the discussion of the parallel between the quality-quantity tradeoff described here and the well-known bias-variance tradeoff in the statistics literature, and (ii) the synthesis of analytic derivations, experiments on synthetic data where the "true" fitness function is known, and applications on seven real different datasets. My two major comments are as follows:

1. The authors claim that the error due to bias "reveals how much of the epistatic interactions shaping the fitness landscape are left unmodeled." I am not convinced that this is true in general. In particular, they give an example (lines 190-194):
Assume first that we use the independent-site model for fitness prediction. If the 'true' fitness landscape shows no epistasis, this model is exact (up to statistical fluctuations due to the finite amount of training data, taken care of by σ2), and the bias vanishes. Therefore, a non zero bias would signal the presence of epistatic interactions between residues not captured by the simple model used for predictions.

I do not follow this logic. With a 'true' linear fitness landscape, bias will occur if the empirical frequencies of the residues at a position are not perfectly correlated with the additive fitness effects of the corresponding substitutions at that position.

To be concrete, imagine at a fixed position the fitness effect of amino acid A is greater than the fitness effect of amino acid B, but in the MSA B is more common than A. The model, which is based on the assumption that empirical frequencies correlate with the fitness effect, will infer that B is more fit than A, incurring an error that has nothing to do with epistasis. (This type of scenario could occur for many reasons: perhaps A confers some disadvantage in translation in some organisms, perhaps A is more present across the whole protein family but the full MSA we have is a biased subsample based on what we have sequenced where B happens to be more common, perhaps there is a population genetic reason for the relative abundance of B.)
Figure 5C shows a trend that as the model includes more epistatic terms (K), the bias decreases. This suggests that in these examples the bias (in particular for low K) might be due to unmodeled epistatic interactions. Nonetheless, I don't think it is fair to in general equate bias, which could be caused by a number of forms of model misspecification, with unmodeled epistasis.

Reviewer 2 is right to stress that our statement quoted above is correct in an idealized setting, where the only source of bias is the mismatch between the model and the ground-truth fitness landscape. We agree that, in reality, many sources of biases can be present. In particular, biases can come from sampling e.g. some organisms are more studied and sequenced than others, or from the unclear relationship between sequence statistics and the specific biochemical property measured through in vitro mutation scans.
The source of bias we focus on in our work, while being important, is therefore not unique. In the new manuscript, we make clear that unmodeled epistasis is a contribution to the bias, among others. Changes have been brought at different points of the main text to emphasize this point, see in particular lines 195-201 and 496-500. We hope Reviewer 2 will find the new presentation fairer.

2. The authors propose an empirical rule for defining a cutoff (d_snr) to determine which sequences to include in the MSA for fitness effect prediction with a Potts model. I suggest that the authors more cohesively present the predictive performance of the model when d_snr is used. To this end:

(i) Please address how the SNR threshold of 3 was chosen (presumably fit on these 7 families; perhaps better to fit on a subset of the families and evaluate on the other families; having only 7 families makes this difficult),

The SNR threshold of 3.0 was not optimized on our data set but was arbitrarily chosen as a "three-sigma rule of thumb". Prompted by the reviewer's comment, we ran a systematic analysis of how different SNR thresholds affect the delta performance of our statistical models. The results, summarized in the figure below, show that an SNR of 3 is in fact not optimal, and an SNR threshold of 4.0 gives better performances on the 7 families considered here. We have modified the man text accordingly, and have added these results to the manuscript in a new supplementary figure.

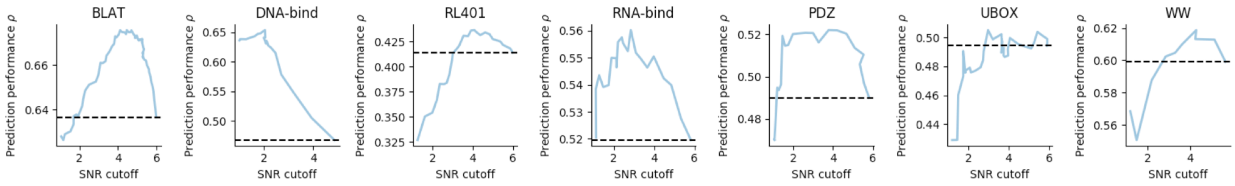

(ii) produce the analog of Figure 4E for the d_snr cutoffs and include it in Figure 4 (it is hard to deduce this from Fig 4F), and

We added this analysis to the new supplementary figure as suggested by the reviewer.

(iii) reference Supplementary Figure 5 in the section "Cutoff for optimal focusing can be reliably predicted from heuristics" (Line 324).
We added the reference suggested by the reviewer.

Additional comments:

3. The choice of “quality” to describe which sequences are best to use for a specific task is a bit odd. Generally, one thinks of a “quality” MSA as one containing a diverse set of well-aligned sequences. In the paper, a high-quality MSA refers to one where the degree of similarity between the sequences and a given wild type is optimal for some downstream task. Instead of describing the tradeoff as “quality” versus “quantity”, perhaps it is better to frame it as “relevance” versus “quantity”. It is not that the MSA becomes higher quality when you remove dissimilar sequences, but rather the sequences that remain are the most relevant for the prediction task.

We agree with Reviewer 2 that the meaning of the vocable quality in our manuscript was relative and not absolute: it referred to the similarity to the wild-type (wt) sequence. In other words, a sequence is intended to be “good” if its content is not too different from wt. To avoid any confusion, following the recommendation of Reviewer 2, we now use “relevance” instead of “quality” throughout the manuscript (including the author summary).

4. Equation 1, which expresses the mean squared error as the sum of bias and variance, is difficult to parse due to notation. The authors use E-hat-i-a to express both the (i) the predicted fitness when amino acid a is at position i and (ii) the expectation of this quantity under repeated predictions with different subsets of data (with same quality and quantity statistics). A literal reader would conclude that the variance is always zero. Please improve the notation.

In the previous manuscript, we used the notation [.] to denote the average over different subsets of data of E-hat_{i,a} and make the difference between this estimator and its mean value. The [.] notation was defined through a Latex \newcommand, which, for some reason, did not compile properly. We apologize for this inconvenience, which made notations very confusing. We have solved this technical issue in the new manuscript.

5. Please add a couple sentences about lattice proteins (e.g. how are the folds determined) to the main text to make the section more self-contained. (Line 209)

We have added a sentence explaining how the wildtype sequence and native fold were chosen.

6. The subsection “J_0 can be inferred without any knowledge of the true fitness landscape” (Line 265) describes how to estimate J_0 using rho, the predictive performance. Computing rho requires the measured fitness, which is something about the fitness landscape. The title of the subsection does not make sense.

We agree with Reviewer 2 that this title was misleading, as measurements are obviously needed to find J_0. We meant “... without **explicit** knowledge …”. To avoid confusion, we have changed the title into “J_0 can be inferred from mutational scan data“ (line 270).

7. Fig 4E + Lines 313-323: To what extent is the improvement in performance between MSA(full) and MSA(d_opt) correlated with the difference in average Hamming distance to the wt in MSA(full) and MSA(d_opt)? (Sup Fig 5 suggests that it is.)

We thank the reviewer for this suggestion. As "relevance" is closely related to the mean Hamming distance to the wild-type, there might be a correlation between the increase in performance and the difference in the mean distance before and after the focusing procedure. However, while we indeed observed a positive relation between the relative increase in performance and the relative decrease in mean Hamming distance, the correlation between the two quantities was not significant (Pearson R=0.68, P=0.09; Spearman corr=0.14, P=0.75).

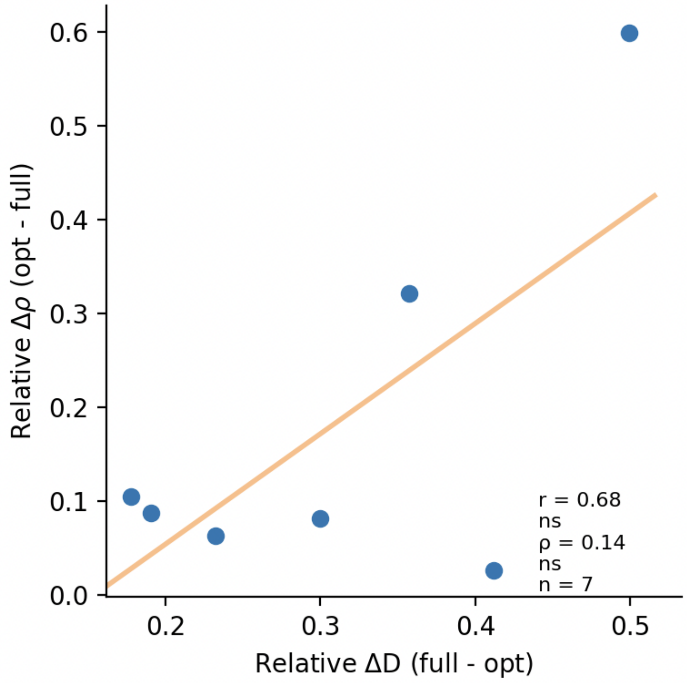

8. Fig 5C: how might you account for the non-monotonicity in the curves (in particular WW)?

The estimation of J0 is done by minimizing the Spearman correlation between the sum of bias and variance and the prediction performance of the statistical model. Since multiple values of J0 can correspond to the same maximum Spearman correlation, we take the mean J0 across those that maximize the correlation as our estimation. As a result of this redundancy, some plots might look non-monotonous because the interval of J0s that maximizes the correlation is larger than the variation of J0 with K. This is especially true In the case of the family WW, which was rightfully indicated by the reviewer as an anomaly in the decreasing trend of J0 with K.

9. Appendix A: Please justify the approximation <p>(1-<p>) = <p>. Please explain where the (1-k_i) term in equation (20) comes from. How does the reweighting procedure affect the estimate (21)? Are the p_i(a) computed with respect to the weights on the sequences within the MSA?

The p_i(a) are computed taking into account the weights of the sequences. We have largely rewritten Appendix A in Supporting Information to carefully explain the derivation of the variance, first in the independent-site case, then in the presence of couplings. The presence of the term (1-k_i) is now much easier to understand, compare the first and second lines of Eq. (26) in Supporting Information.

10. AlphaFold predictions are known to be sensitive to the input MSA; in CASP15 some methods found improved predictions by changing the way MSAs were generated (see e.g. Elofsson, 2023, Progress at protein structure prediction, as seen in CASP15). Perhaps this is worth mentioning to further your point that the selection of sequences is an important part of a task.

We thank Reviewer 2 for making this point. We have included it in our Discussion (lines 554-557), with a pointer to the reference.

11. What value of K was used in producing Supplementary Fig 5? Please include in the caption.

We clarified that the independent model was used to obtain the results of Supp. Fig. 5 in the revised figure caption.

12. I could not find code to reproduce the results in the paper.
We now provide the code and data to reproduce the main figures of the paper on biological proteins at this link: https://www.dropbox.com/scl/fi/829vkm6090m7qm8hqwoc9/Code.zip?rlkey=5z2960xkiq6xr43uxf0updfrm&dl=0. The code to generate lattice proteins was taken from Jacquin et al. 2016, PLoS Comp Bio.

Typos/style:
1. The caption of Fig 1 refers to a grey dashed line, but there is no such line in the figure.
2. Line 128 and caption of Fig 2: wrong character for <
3. Don’t start sentences with mathematical characters (e.g. Lines 181, 205)
4. Mismatched quotes; use `` instead of “ for left quotes in LaTex (e.g. Line 307, 477)
5. andWe→,andwe(Line351)
6. decrease → decreases (Line 367)
7. Fig 4A: add label to y-axis
8. Equations 13 and 14: some rendering issues? The = missing?
9. grounth → ground (Line 763)

We thank the reviewer for finding these typos. We corrected them in the revised manuscript.
